# Static and Dynamic Stiffness of Reinforced Concrete Beams Strengthened with Externally Bonded CFRP Strips

**DOI:** 10.3390/ma14040910

**Published:** 2021-02-14

**Authors:** Michał Musiał, Tomasz Trapko, Jacek Grosel

**Affiliations:** Faculty of Civil Engineering, Wroclaw University of Science and Technology, Grunwaldzki 11, 50-370 Wroclaw, Poland; tomasz.trapko@pwr.edu.pl (T.T.); jacek.grosel@pwr.edu.pl (J.G.)

**Keywords:** CFRP, reinforced concrete, strengthening, stiffness, modal testing

## Abstract

This paper presents experimental investigations of reinforced concrete (RC) beams flexurally strengthened with carbon fiber reinforced polymer (CFRP) strips. Seven 3300 mm × 250 mm × 150 mm beams of the same design, with the tension reinforcement ratio of 1.01%, were tested. The beams differed in the way they were strengthened: one of the beams was the reference, two beams were passively strengthened as precracked (series B-I), two beams were passively strengthened as unprecracked (series B-II) and two beams were actively strengthened as unprecracked (series B-III). Moreover, the strengthening parameters differed between the particular series. The parameters were: CFRP strip cross-sectional areas (series B-I, B-II) or prestressing forces (series B-III). The beams were statically loaded, up to the assumed force value, in the three-point bending test and deflections at midspan were registered. After unloading the beams were suspended on flexible ropes (the free-free beam system) and their eigenfrequencies were measured using operational modal analysis (OMA). The static measurements (deflections) and the dynamic measurements (eigenfrequencies) were conducted for the adopted loading steps until failure. Static stiffnesses and dynamic stiffnesses were calculated on the basis of respectively the deflections and the eigenfrequencies. The qualitative and quantitative differences between the parameters are described.

## 1. Introduction

One of the basic ways in which civil engineering structures can be diagnosed is by observing their response to external impacts of usually known magnitude. The observations supply many data important from the engineering point of view. The parameter usually traced during such investigations is displacement. The intensive development of non-destructive structural diagnostics in recent decades [1,2] has resulted in new attractive methods of testing building structures, elements and materials. One of such methods is the operational model analysis (OMA) [3]. Using OMA one can determine certain dynamic parameters (e.g., eigenfrequencies, eigenforms and damping) of a structure on the basis of its response to random excitations, without measuring the parameters. This method is applicable to traditionally understood static loading modes (light wind blows, vehicle traffic close to the structure)—the metrological precision of the measuring instruments currently used guarantees acquiring vibrations caused even by such a small load. OMA is, therefore, a method that is widely used in both statically and dynamically loaded structures. For many years this and similar methods have been successfully used to diagnose civil structures [4,5,6], their models tested in laboratory conditions [7,8], building elements [9,10], concrete as a material [11,12,13] and even concrete in its maturation phase [14].

As part of the present research the authors decided to compare static measurement results, in the form of deflections, with dynamic measurement results in the form of eigenfrequencies. These quantities are not directly comparable. Knowing the force or the eigenfrequency one can determine the flexural stiffness of a beam on the basis of the measured deflection. According to the convention adopted in the literature [9,15,16,17,18,19,20], the considered quantities are called: static stiffness and dynamic stiffness, respectively. In order to calculate them one can use the basic relations from strength of materials and structural dynamics. The relations are quoted and discussed later in this paper.

The dynamic characteristics of the system provide information on the structure condition. The conclusions about the structure—in particular its stiffness—can be drawn by monitoring its dynamic test characteristics. This is a practically viable approach to any structure regardless of its static or dynamic load.

**Figure 1:**
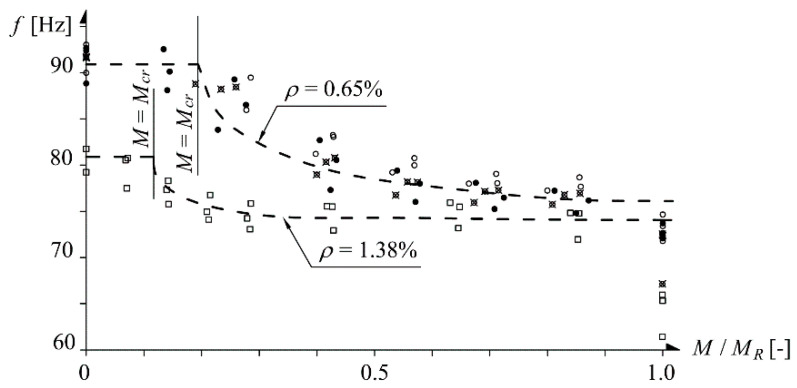
Eigenfrequencies f versus loading for beams with different tension reinforcement ratio ρ [9].

The problems mentioned above become even more interesting when one considers that perpendicular cracks occur in all typical rationally designed reinforced concrete structures subjected to bending. Such cracks appear once the cracking moment is exceeded. Cracks are the main cause of flexural stiffness degradation, and so they have an influence on, i.a., deflections and eigenfrequencies—the parameters traced during the tests described in this paper. Exemplary graphs showing the influence of the loading of beams on their eigenfrequencies are presented in Figure 1 [9]. One can see a fall in eigenfrequencies after the cracking moment is exceeded. The fall has a different character depending on the reinforcement ratio (0.65% and 1.38%).

**Figure 2:**
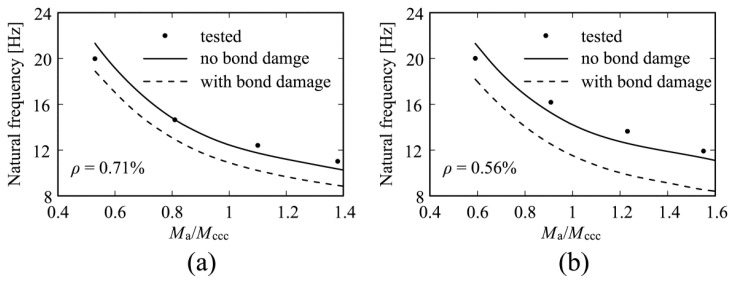
Eigenfrequencies versus loading for beams with tension reinforcement ratio ρ of (**a**) 0.71% and (**b**) 0.56%.

Another cause of stiffness degradation is steel-concrete bond damage. Its consequences were examined in, i.a., [18]. Figure 2 shows graphs of eigenfrequency versus loading (on the basis of [18]). The effect of bond damage on the eigenfrequencies is visible (the dashed lines) in the case of the calculated values.

**Figure 3:**
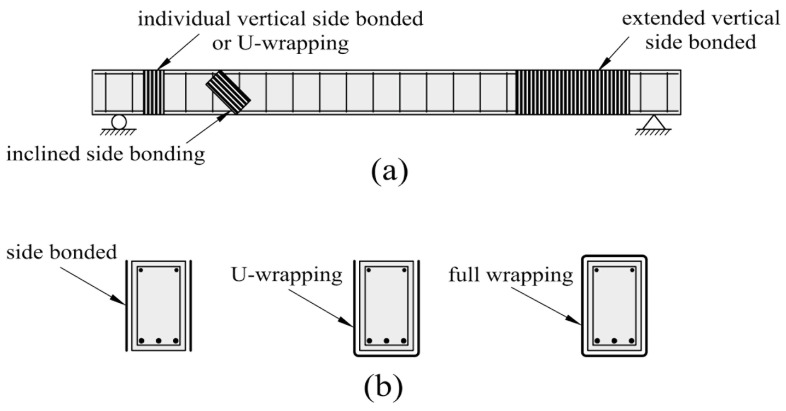
Illustration of use of fiber reinforced polymer (FRP) for shear strengthening in reinforced concrete (RC) beams: (**a**) typical FRP strengthened beam, (**b**) various FRP schemes

Although the paper focuses on strengthening flexure (what is sufficient from the point of view of the aim of the presented studies), an equally important element of system strengthening relates to additional external shear reinforcement. Usually, deficient reinforced concrete (RC) beams suffer also from shear problems due to insufficient shear reinforcement (stirrups). Composite shear strengthening is not the object of this paper. It is the authors’ opinion that the method for increasing the shear capacity of beams should be briefly presented here. The most common and effective ways of shear capacity improvement are shown in Figure 3 (prepared on the basis of [21]). Moreover, additional vertical reinforcement near the supports improve the anchoring conditions of the external longitudinal reinforcement.

In case of T-shapes beams the application of the externally bonded shear reinforcement results in additional anchoring problems. This issue was discussed in [22]. The anchoring strengthening methods were proposed. An additional shear strengthening method consists of applying of slightly reinforced thin U-shaped cementitious mortar jacketing [23]. The method is appropriate even in case of heavily damaged beams.

It should be noted that the tests and analyses presented in this paper have rather a qualitative character. Their aim is to show the different character of the behavior of variously strengthened beams in terms of static and dynamic stiffness. The results of the investigations can be used in, e.g., the diagnosis of reinforced civil engineering structures by means of OMA. It should be emphasized that currently there is no research on the static and dynamic predispositions (in this case, deflections and eigenfrequencies) of structures strengthened with carbon fiber reinforced polymer (CFRP) strips. Currently, dynamic measurements are used mainly to identify damage to strengthened beams [19,24,25].

The presented studies are the attempt to connect the following contemporary methods: the structural diagnostics (OMA) and the strengthening of the structures with CFRP materials. On the basis of the literature studies and the authors’ own expertise it can be stated that certain issues should be recognized, verified and compared.

## 2. Aim and Scope of this Research

The aim of this research was to determine the effect of the degradation of flexural stiffness (caused by perpendicular cracks)—one of the basic parameters of reinforced concrete beams. The tested RC beams were variously strengthened with Sika CarboDur M CFRP strips [26]. The stiffness of the beams was determined on the basis of: (1) deflections (static stiffness) and (2) eigenfrequencies (dynamic stiffness).

Seven beam members of the same design were subjected to the tests. Beam B-0 served as the reference and was used mainly in experimental determination of the resistance to bending. The other beams were variously strengthened with CFRP strips, as described in detail further in this paper. The beams were subjected to loading in the three point bending test. The deflections (under loading) and eigenfrequencies (after each unloading) were registered. The author decided to study the eigenfrequencies of the beam after unloading in order to separate the effect of advancing cracking (resulting in a decrease in stiffness) from the effect of the increasing mass (the increment in inertia). As shown in [27] the factors act synergistically. Thus, it can be formally stated that in the case of deflections the effect of the load on the static stiffness was determined, whereas in the case of eigenfrequencies the effect of the loading history on the dynamic stiffness was determined. The dynamic testing was conducted using operational modal analysis.

Additionally, the strength and deformability of the reinforcing steel and the concrete were determined. The properties of the CFRP composites used to strengthen the beams were taken from the manufacturer’s specifications [26].

## 3. Materials and Methods

### 3.1. Test Specimens

**Figure 4:**
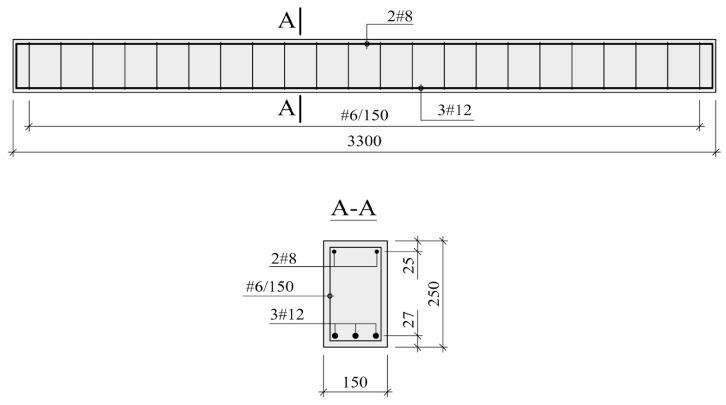
Beam—test specimen (dimensions in mm).

The beams for the tests were ordered from a prefabrication plant. They all had an identical structure (Figure 4). They were designed to minimize the scale effect. Their outer dimensions were: 3300 mm × 250 mm × 150 mm.

The specimens were made of natural aggregate concrete. It was specified that the beams’ concrete should be characterized by the average E-modulus of 30 GPa, which corresponds to the E-modulus of the concretes most often used in practice. Considering that the E-modulus of concrete is the key parameter having a bearing on the flexural stiffness of beams, no specific requirements as to the other parameters of the concrete were made. Steel B500SP was used for the longitudinal reinforcement, while steel B500A was used for the stirrups. The nominal characteristic yield strength of the two steels is *f*_yk_ = 500 MPa. The steel used for the longitudinal reinforcement is characterized by ductility grade C, while the steel of the stirrups has ductility grade A [28]. The tension reinforcement ratio amounted to 1.01%. This value can be recognized as economically viable, rational and commonly used in practice.

### 3.2. Materials and Their Properties

As regards the properties of the concrete the following were experimentally determined:

- Mean compressive strength, on cylinders 150 mm in diameter and 300 mm high—*f*_cm_,
- Mean E-modulus, on cylinders 150 mm in diameter and 300 mm high—*E*_cm_,
- Mean splitting tensile strength, on 150 mm cubes—*f*_ctm,spl_.

In the case of the steel, the following were determined through the classical steel bar tensile test:

- Mean yield strength—*f*_ym_,
- Mean E-modulus—*E*_sm_,
- Mean tensile strength—*f*_yt_.

The strength and deformation parameters of the CFRP composite used to strengthen the structure were taken from the CFRP strip manufacturer’s specifications [26]. The parameters were:

- Mean tensile strength—*f*_f_,
- Mean E-modulus—*E*_f_,
- Minimal rupture strain—*ε*_fu_.

All the presented properties of the materials used in the tests are shown in Table 1.

### 3.3. Test Stand

**Figure 5:**
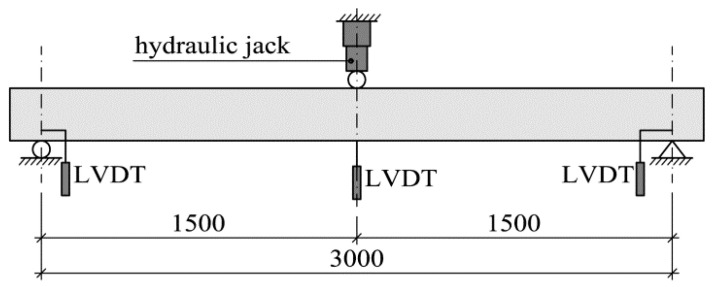
Schematic diagram of tests stand for static measurements (dimensions in w mm).

**Figure 6:**
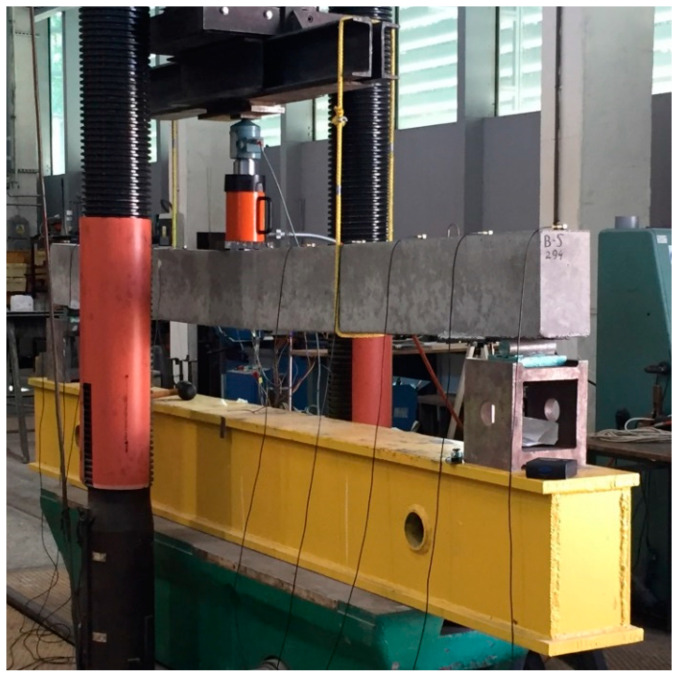
Test stand for static measurements.

As already mentioned, two types of measurements: static (the measurement of deflection under loading) and dynamic (the operational modal analysis of the beam after unloading) were conducted. Loading was applied in the three-point bending test. Deflections were measured at midspan (under the force) using an inductive sensor with an accuracy of 0.001 mm. In addition, deflections were monitored at the supports in order to record their potential subsidence. Figure 5; Figure 6 show a schematic diagram and a photograph of the test stand for static measurements.

In the case of dynamic tests, the simply supported beam system was abandoned due to difficulties in reproducing it in the tests. Previously, when realizing the pin support, a problem consisting in support point detachment (uplift) had always been encountered [9,11]. This is due to the low amplitudes of the vibrations during the operational modal analysis, which are impossible to eliminate in the case of the steel components making up the conventional pivot bearing. Another problem can be posed by the compliance of the supports, considerably affecting the dynamic response of the structure, which was exhaustively analyzed in [29].

**Figure 7:**
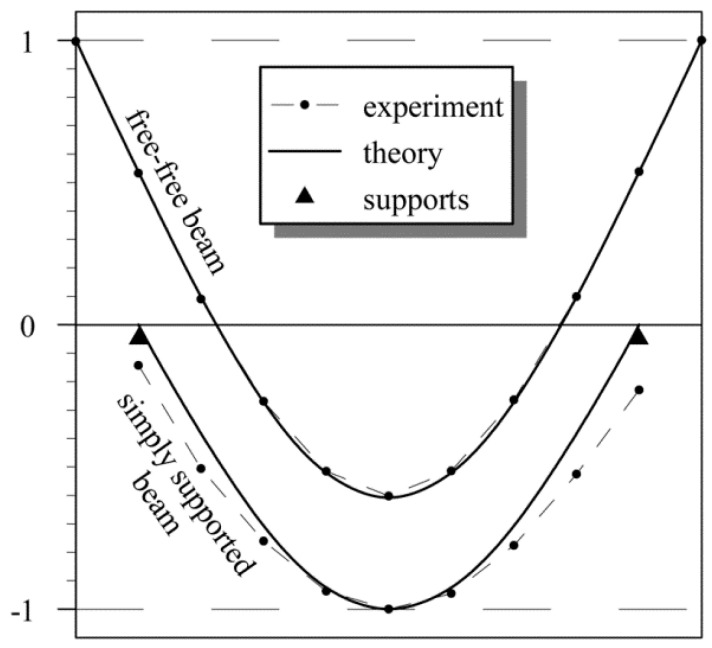
Theoretical and experimental first eigenforms for two loading diagrams [11].

As evidenced by the first eigenforms acquired with the two experimental setups, it is much more advantageous for dynamic tests to use the free-free beam arrangement. The eigenforms are shown in Figure 7 [11]. It can be seen that in case of the simply supported beam, movements are registered on the support base despite the use of the bilateral screw-bearing. The beam’s first acquired eigenform reflects our earlier theoretical predictions. The discrepancy between the theoretical model and measurement results was negligible.

**Figure 8:**
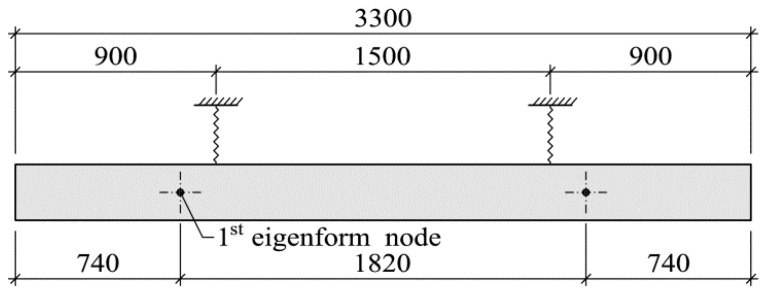
Schematic diagram of the test stand for dynamic measurements (dimensions in mm).

**Figure 9:**
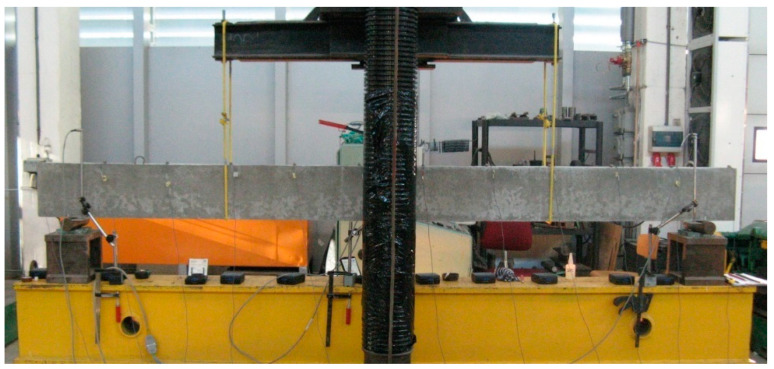
Test stand for dynamic measurements.

Suspension by means of two flexible ropes was used to realize the free-free system for the tests. A schematic diagram and a photograph of the test stand are shown in respectively Figure 8 and Figure 9. In [30] it is recommended for the suspension points to be located in the nodes of the observed eigenform. For practical reasons the tested beams were suspended in points shifted relative to the nodes of the first eigenform towards the middle of the specimen. However, previously it had been ascertained that this location of the suspension ropes had little effect on the results (eigenfrequencies and eigenforms).

### 3.4. Instrumentation and Registration of Measurements in Operational Modal Analysis

#### 3.4.1. Theoretical Foundations

In modal analysis the dynamics of a structure are described by modal parameters, i.e., eigenfrequencies, eigenforms and modal damping [31,32]. Knowing the modal characteristics one can identify other parameters of the structure [33]. One can also trace the variation of the dynamic characteristics over time and on this basis infers about the degree of degradation of the structure [34]. The modal characteristics of a structure can be investigated in two ways. One of the ways is the classical modal analysis the called experimental modal analysis (EMA). It consists of simultaneously measuring the vibration exciting forces and the system response to this excitation. The application of EMA to civil structures entails measurement difficulties mainly due to the necessity of exciting massive and rigid structures and when modal hammers are used close to the measuring point, transducers’ overload can arise. Operational modal analysis (OMA) is devoid of the above drawbacks. It consists of measuring the response of a system without measuring the excitation force. The source of vibrations is a natural (operational) load. In comparison with EMA, this method requires longer measurements and the use of transducers of much higher sensitivity since the levels of vibration arising from operational excitations are much lower than those due to controlled excitations.

The algorithms used in OMA can be divided into two basic types: operating in the time domain and operating in the frequency domain. The first group includes algorithms based on the dynamic state equation and its stochastic subspace identification (SSI). The second group comprises algorithms based on discrete system response decomposition into a sum of the responses of SDOF systems in the frequency domain–frequency domain decomposition (FDD) [35].

The basic assumptions that the analyzed system must satisfy are:

- the linearity and time-invariance of the system parameters—a linear time-invariant (LTI) system structure,
- small damping,
- well separated eigenvalues,
- the structure excitation can be estimated as a white noise process.

In such a system the dependence between structure excitation x(t) and response y(t) can be written as follows:

(1) $$[G_{yy}\left(j\omega \right)]={\left[H\left(j\omega \right)\right]}^{*}[G_{xx}\left(j\omega \right)]{\left[H\left(j\omega \right)\right]}^{T}.$$

The symbols represent: [ ]—a matrix, ${\left[\;\right]}^{T}$—a transposition and ${\left[\;\right]}^{*}$—a complex conjugate. $\left[G_{yy}\left(j\omega \right)\right]$ and $\left[G_{xx}\left(j\omega \right)\right]$ are matrices of the spectral power density of respectively the input signal and the output signal. Vectors x(t), y(t) are usually of different size—hence matrices $\left[G_{\ldots }\left(j\omega \right)\right]$ are square, while [H(jω)] is a rectangular spectral transmittance matrix that can be expressed by the formula:

(2) $$\left[H\left(j\omega \right)\right]=\sum_{i=1}^{l}\left(\frac{\left[R_{i}\right]}{j\omega -\lambda_{i}}+\frac{{\left[R_{i}\right]}^{*}}{j\omega -\lambda_{i}^{*}}\right),$$

where l stands for the number of considered modes.

The quantities in formula (2) can be calculated from the relation:

(3) $$\left[R_{i}\right]=\psi_{i}a_{i}^{T},$$

where $\psi_{i}$ is an eigenvector corresponding to the *i*-th eigenfrequency, while vector $a_{i}$ is a vector of the fraction coefficient of the *i*-th eigenform (modal participation vector) and $\left[R_{i}\right]$ is a modal residue matrix corresponding to the *i*-th eigenfrequency.

Substituting (2) into (1) and performing transformations and decomposition into partial fractions one gets:

(4) $$[G_{\mathrm{yy}}\left(j\omega \right)]=\sum_{i=1}^{l}\left(\frac{\left[A_{i}\right]}{j\omega -\lambda_{i}}+\frac{{\left[A_{i}\right]}^{H}}{j\omega -\lambda_{i}^{*}}+\frac{{\left[A_{i}\right]}^{*}}{-j\omega -\lambda_{i}}+\frac{{\left[A_{i}\right]}^{T}}{-j\omega -\lambda_{i}^{*}}\right).$$

Consistently with the white noise excitation assumption, matrix $\left[G_{xx}\left(j\omega \right)\right]$ is constant matrix $[G_{xx}\left(j\omega \right)]=[G_{xx}]$. On this basis one can determine the matrix of residues:

(5) $$\left[A_{i}\right]=\frac{\left[R_{i}\right]\left[G_{xx}\right]{\left[R_{i}\right]}^{H}}{2\sigma_{i}},$$

where symbol ${\left(\;\right)}^{H}={\left[{\left(\;\right)}^{*}\right]}^{T}$ stands for the Hermitian conjugate.

Using the small damping assumption and relation (3) one gets the relation:

(6) $$\left[A_{i}\right]=\left[R_{i}\right]\left[G_{xx}\right]{\left[R_{i}\right]}^{T}=\psi_{i}a_{i}^{T}\left[G_{xx}\right]a_{i}\psi_{i}^{T}=d_{i}\psi_{i}\psi_{i}^{T},$$

where, as previously, $\psi_{i}$ is the *i*-th eigenvector, $a_{i}$ is a vector of constant coefficients for the *i*-th eigenform and $d_{i}$ a certain constant. Then the spectral power density matrix can be written as:

(7) $$[G_{yy}\left(j\omega \right)]=\sum_{i=1}^{l}\left(\frac{d_{i}\psi_{i}\psi_{i}^{T}}{j\omega -\lambda_{i}}+\frac{d_{i}\psi_{i}^{*}\psi_{i}^{H}}{j\omega -\lambda_{i}^{*}}\right),$$

where summation is performed only over the modes having a significant effect on the specific frequency (usually no more than two components).

The response matrix can be decomposed using singular value decomposition (SVD) to this form:

(8) $$[G_{yy}\left(j\omega_{i}\right)]=\left[\Phi_{i}\right]\left[S_{i}\right]{\left[\Phi_{i}\right]}^{H},$$

where matrix $\left[S_{i}\right]$ is a diagonal singular value matrix and subscript “*i*” denotes decomposition for a specific discrete frequency. Matrix $\left[\Phi_{i}\right]$ is a unitary matrix containing vectors proportional to the eigenvectors. From matrix $\left[S_{i}\right]$ one can calculate the eigenfrequencies.

#### 3.4.2. Instrumentation

**Figure 10:**
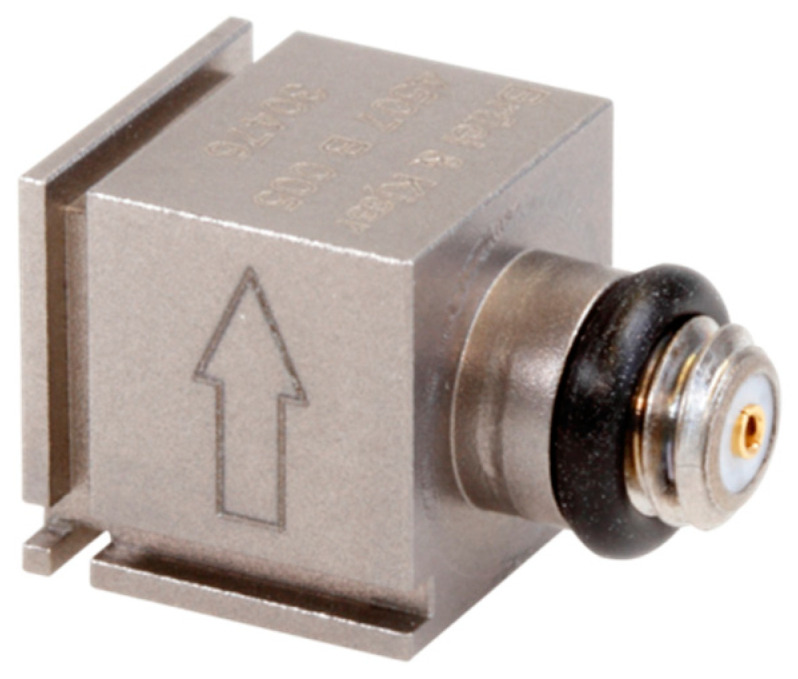
Piezoelectric accelerometer ThetaShear 4507B-005.

Miniature piezoelectric accelerometers 4507B-005 (Figure 10) made by Brüel and Kjær (Nærum, Denmark) were used for measurements. The accelerometers are characterized by high sensitivity and small weight. They incorporate preamplifiers and a transducer electronic data sheet (TEDS).

The most important specifications of the transducers are:

- High sensitivity—1000 mV/g,
- Frequency range of 0.4 Hz to 6 kHz,
- Measuring range up to 70 g (RMS),
- Low noise level—the maximum residual noise level (RMS) in the specified frequency range amounts to 150 μg,
- Small weight—4.8 g,
- Mountable in three directions by means of special plastic clips glued to the structure.

Alternatively, seismic accelerometers (e.g., type 8340) could be used. Such accelerometers are characterized by a much higher sensitivity (10,000 mV/g) and lower noise levels (25 μg), but their weight (775 g) can affect the measurement results.

**Figure 11:**
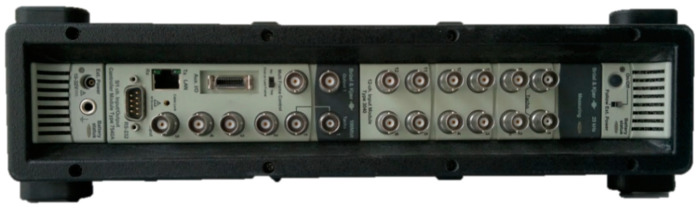
Brüel and Kjær PULSE 3560-C unit.

The accelerometers were connected to a PULSE 3560-C unit (Figure 11).

The specifications of the PULSE 3560-C unit most important for the measurements are:

- Up to 17 accelerometers can be connected to perform simultaneous cophasal measurements,
- Measured signal frequency range of up to 25.6 kHz,
- Measuring modules’ dynamic range of up to 160 dB,
- Input voltage range without distortions (the noise level of 4 μV at the most),
- Maximum peak voltage—10 V, linearity ±0.03 dB at 120 dB,
- Detection and signaling of wiring damage,
- Signaling of overloads in all the channels (input systems), displayed by both the hardware and the software.

The frequency domain decomposition (FDD) identification algorithm and the Artemis software by SVS (Denmark) were used in the investigations [36]. Twelve measuring points were designated on the top surface of each of the beams. The points were located in the longitudinal vertical symmetry plane and spaced at the same distance. The outmost measuring points were located at the edges of the beams. Tests of eigenfrequency identification effectiveness depending on the kind of excitation were carried out. Since the operational excitations occurring in the laboratory hall are small—there are no wind gusts and no vibrations induced by vehicles passing nearby—the measurement would have to last very long in order for vibration inducing incidents (e.g., a pedestrian passing by, an acoustic excitation) to occur. Therefore it was decided to excite the beam by striking it (its top surface) gently with a rubber hammer. Care was taken to avoid any regularity in the excitation by varying the place of striking, the force of striking and the intervals between strikes. Due to this the duration of vibration recording for a single eigenfrequencies determination was reduced from over 30 to 1 min. At the same time the quality of the excitation was good enough to identify the eigenfrequencies.

#### 3.4.3. Results

**Figure 12:**
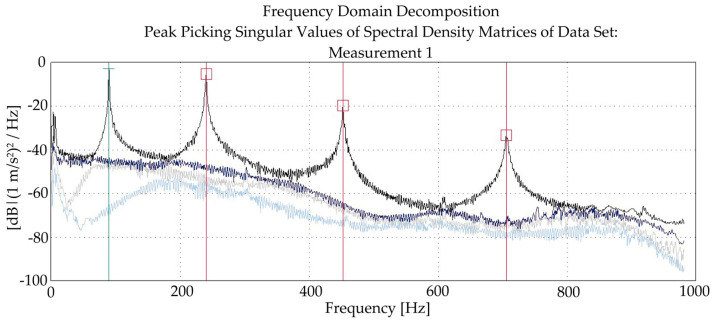
Singular values of spectral density matrices.

An exemplary result of the measurements is shown in Figure 12. It is a decomposition of the singular values of the spectral density matrices in the analyzed frequency range for beam B-II-1 after 10 kN loading. The red cursor lines represent the found eigenfrequency values (occurring in the maxima of SVD no. 1). The identified eigenfrequencies were: 89.5, 240.5, 452.5 and 706 Hz.

### 3.5. Investigative Procedure and Strengthening of Beams

Beam B-0 was the reference used to determine flexural strength without CFRP reinforcement. The investigative procedure in this case was limited to static measurements. Beam B-0 was set up on supports and loaded until failure in the three-point bending test. The deflection at the midspan was recorded.

In the case of the reinforced beams, a combination of two types of measurements, i.e., static measurement and dynamic measurement, was used. An operational modal analysis of the debuting beam (before loading) was carried out. Then the beam was set up on supports and loaded at the initial rate of 5 or 10 kN. The load was maintained until deflections stabilized. After unloading the beam was lifted on the flexible ropes and subjected to OMA. Then the beam was again set up on the supports and loaded with the intensity 5 or 10 kN higher than in the previous step. The further post-unloading operations were executed as in the previous cycle. The above sequences were performed until the beam failed. The loading step of 5 or 10 kN was selected depending on the rate of eigenfrequency changes.

The following types of strengthening were applied:

(1) Passive strengthening of precracked specimens with no injection of perpendicular cracks, loaded with the force of 45 kN (amounting to about 80% of the load capacity)—beams B-I-1 and B-I-2;
(2) Passive strengthening of the debuting specimens—beams B-II-1 and B-II-2;
(3) Active (prestress) strengthening of the debuting beams—beams B-III-1 and B-III-2.

**Figure 13:**
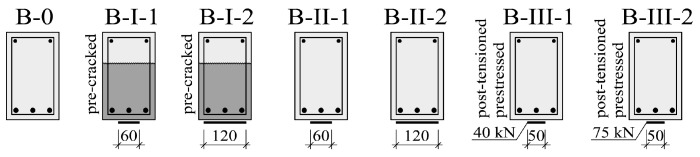
Cross sections of tested beams.

The strengthening intensity was varied within the B-I, B-II and B-III series. The debuting beams (series B-II and B-III) represent a practical case of strengthening after the injection and bonding of cracks caused by mechanical impacts. The cross sections of the beams are schematically shown in Figure 13. All the beams together with their strengthening parameters are compared in Table 2. In the case of the passively strengthened beams, the parameter was strengthening intensity, defined as a ratio of the CFRP strip cross section to the concrete cross section. As it was mentioned in the introduction the static and the dynamic stiffnesses of the beams are the crucial parameters for these investigations. In order to observe the significant impact of the strengthening on the flexural stiffness, the CFRP stripes of M-type were used. They are characterized with the high Young modulus *E*_f_ (210 GPa). In case of S-type stripes The Young modulus was lower (170 GPa). In the beams B-I-2 and B-II-2 the maximum possible area of the CFRP stripes was applied in a single layer (to avoid considering of additional issues introduced with multilayers application). It is the authors’ conviction that the expected impact of the applied strengthening on the stiffnesses should be significant (as a consequence of the high E-modulus CFRP stripes and the area of them). In case of the beams B-I-1 and B-II-1 50% of the CFRP stripes area was applied for comparison. As a result, the strengthening of the beams seems to be unjustified from the point of view of a load capacity (e.g., the high strength system applied without any additional anchoring members and U-wrapping at the ends). On the other hand, the strengthening system provides the high increase of the flexural stiffness.

In the case of the actively strengthened beams, the parameter was the nominal prestressing force. For beam B-III-2 such a maximum prestressing force was selected so as not to exceed the beam’s bending strength for the top reinforcement under tension. The load capacity was estimated at 11 kNm, which corresponds to the longitudinal compressive force of about 85 kN situated on the axis of the CFRP strip. In the case of beam B-III-1, about 50% of this force (40 kN) was applied.

All the beams were strengthened in the reverse position. Beams series B-I were strengthened after complete unloading. The surfaces of the specimens to be strengthened had been properly prepared by cleaning them of bleeding water, dedusting and degreasing. The CFRP strips were degreased. A system adhesive based on epoxy resins [26] was used to glue the strips to the beams. The specimens were left for 48 h in order for the glue to dry. After this time they were regarded as ready for the tests or for relieving the prestress (B-III).

**Figure 14:**
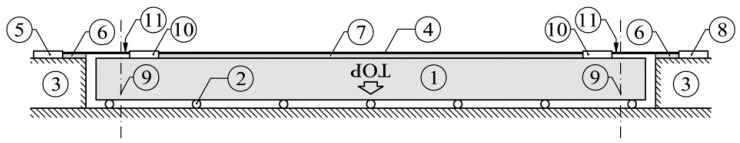
Schematic diagram of stand for prestressing beams series B-III (description in text).

A schematic diagram of the stand for prestressing the series B-III beams is shown in Figure 14. The beams (1) would be placed top down on sleepers (2) between abutments (3). CFRP strip (4) (after fitting and preparing the strip and beam surfaces to be glued) was secured in dead end anchorage (5). The surface of the contact between the strip and the abutments or the beam was properly lubricated with sliding facilitating substance (6) or epoxy adhesive (7). The prestressing force was applied by means of a hydraulic cylinder in live end anchorage (8). After the proper prestressing force (40 or 75 kN) had been reached, anchoring plates (10) were fixed (using an adhesive and steel pins) near the axes of the supports (9). The strip was pressed against the beam and the excess adhesive oozing out from under the strip was leveled. After 48 h of adhesive hardening the prestress was released in the live end anchorage and the strip was cut off (11) immediately behind the anchoring plates. During prestressing the strains in the midsection of beams series B-III were registered by means of electrical resistance strain gauges. On the basis of the strains the distributions of stress were determined. The distributions, in turn, were used to calculate the effective prestressing forces introduced into the beam. The forces were: 28.35 kN and 41.27 kN, respectively.

## 4. Main Test Results 

### 4.1. Static Measurement Results

**Figure 15:**
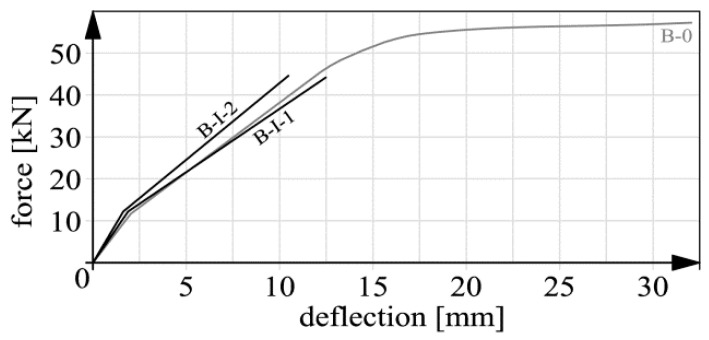
Deflection graphs for beam B-0 and beams series B-I before strengthening.

**Figure 16:**
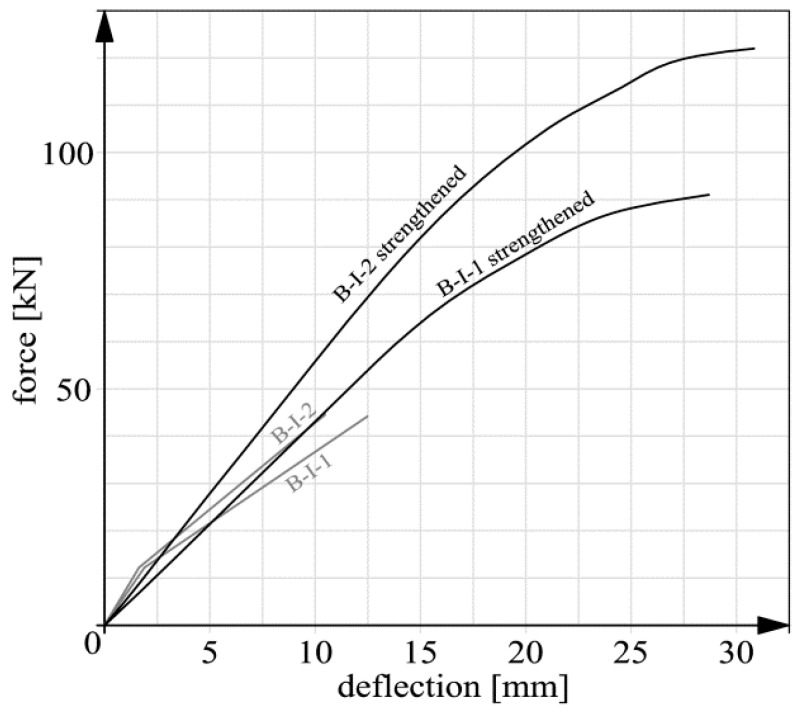
Deflection graphs for beams series B-I before and after strengthening.

**Figure 17:**
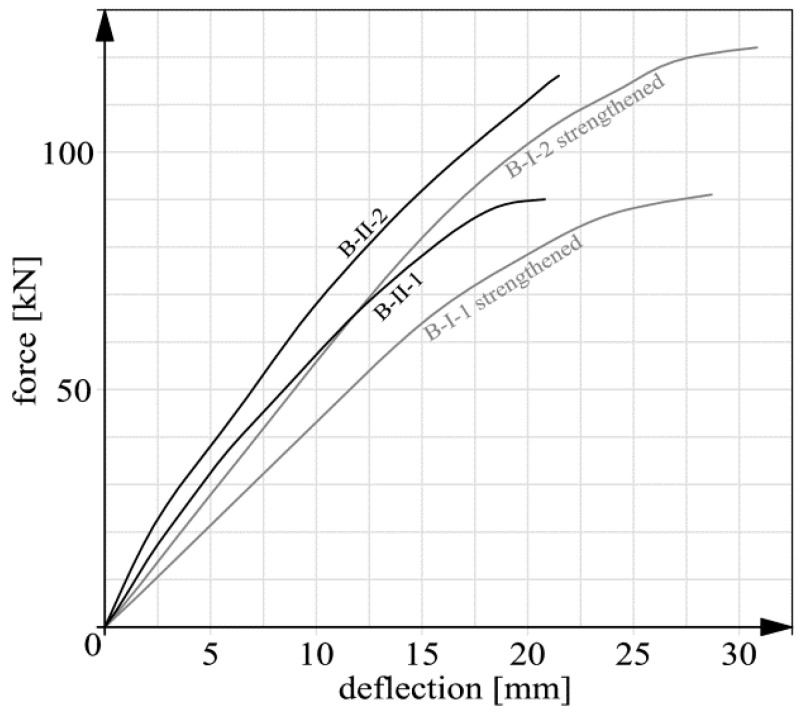
Deflection graphs for beams series B-II and B-I after strengthening.

**Figure 18:**
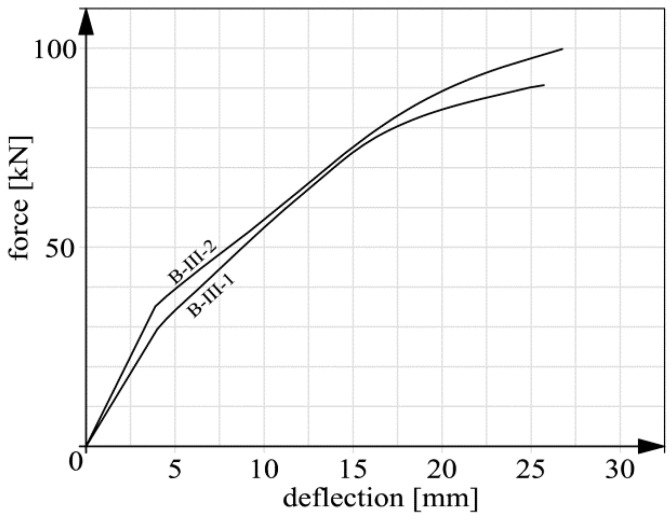
Deflection graphs for beams series B-III.

Basic deflection measurement results are shown in Figure 15, Figure 16, Figure 17 and Figure 18. In the case of beams B-III, the reverse deflections induced by prestressing were omitted. In comparison with the total deflections, their values were very low (about 0.61 and 0.89 mm for respectively beam B-III-1 and B-III-2).

It should be noted that the static behavior (including deflections) of members strengthened with composite materials has been the subject of research (e.g., [26,37,38,39,40]) for many years. This means that the presented graphs (Figure 15, Figure 16, Figure 17 and Figure 18) are not intended to qualitatively contribute to the existing knowledge on this subject. The load–deflection curves (Figure 15, Figure 16, Figure 17 and Figure 18) are included and analyzed in this paper since they were the starting point for further analyses of the static predispositions of the beams, mainly their static flexural stiffness. Table 3 shows the values of the forces, bending moments and deflections for the instants of cracking and failure, respectively.

In the course of the tests the following failure mechanisms were observed:

- beam B-0–yielding of the reinforcing steel;
- beams series B-I, beam B-II-1–debonding of the CFRP strips together with the concrete cover,
- beam B-II-2–premature, abrupt debonding of the CFRP strip in the layer of adhesive, which explains the absence of the inflexion point and the flattening of the force-deflection graph before failure;
- beams series B-III–crushing of the concrete in the compression zone.

It should be noted that the unexpected mechanism of failure of beam B-II-2 had no significant bearing on the results of further analyses since the load capacity of beam B-II-2 (116.01 kN) was close to that of beam B-I-2 (121.92 kN) in which an acceptable mechanism of failure was observed. In case of series B-I and beam B-II-1 the failure was not proceeded with the evident yield point of the tension steel reinforcement (compared to beam B-0–Figure 15). This fact resulted from several issues: the high tensile capacity of CFRP stripes itself, the low thickness of the concrete cover (15 mm) and the lack of additional anchoring members. It can be stated that anchoring was the weakest element of the strengthening system. As a result, the internal tension steel rebars were not activated like in the case of beam B-0. However, some symptoms of the steel yielding, such as for example the flattening of the force–deflection curves, were observed [41]. The exception was beam B-II-2 because of the before mentioned premature debonding.

The key characteristic of the strengthening system used is its effectiveness regarding the ultimate limit state. The effectiveness can be defined as a degree of strengthening, being a ratio of the beam’s load capacity after strengthening to its load capacity before strengthening (the load capacity of reference beam B-0). Other important parameters of the strengthening system concern the deflection and cracking serviceability states. In the case of deflection, a descriptive parameter can be the force at which the arbitrary allowable deflection amounting to the 1/250 span between the axes of the supports (3000 mm), i.e., 12 mm in the considered case. As the cracking parameter the cracking moment was assumed.

**Figure 19:**
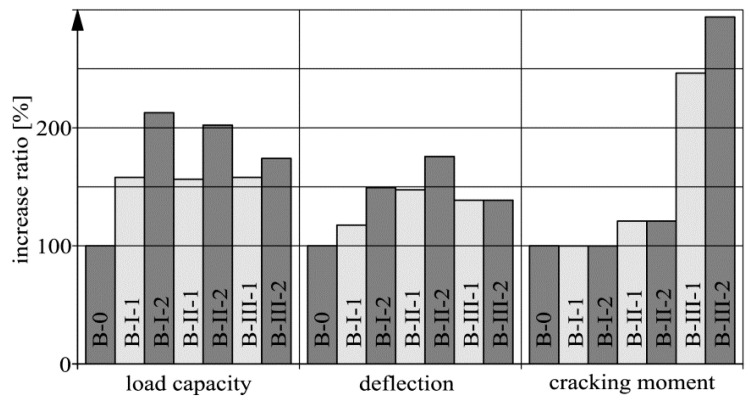
Comparison of selected static parameters of beams.

The above parameters of the serviceability limit state were normalized so that they could be presented together with the degrees of strengthening in one diagram (Figure 19). The normalization consisted in dividing by the result for beam B-0 in the case of the forces associated with the exceedance of the allowable deflection or by the mean for beam B-0 and beams series B-I before strengthening in the case of the cracking moments. Thus one can say that the 100% values correspond to the beam without strengthening.

The results shown in the diagram (Figure 19) were merely a qualitative confirmation of other test results (e.g., [42]), which is presented here as a formality. The largest increase (over twofold) in strength was recorded for the beams passively strengthened with an intensity of 0.448% (B-I-2, B-II-2). The beams passively strengthened with a twice lower intensity were characterized by a strength increase of about 60% (B-I-1, B-I-2). No difference between the behavior of the precracked specimens (series B-I) and that of the unprecracked specimens (series B-II) with the same external CFRP strip reinforcement was found. Even though strips with the smallest cross section (1.4 mm × 50 mm) were used to prestress the beams of series B-III, the measured strength values were close to those obtained for beams B-I-2 and B-II-2 with a slightly larger cross section (1.4 mm × 60 mm). This was due to the use of anchoring plates in beams series B-III, which improved the degree of mobilization of the CFRP strips. The comparable load capacities of beam B-III-1 and B-III-2 are due to the low mobilization of the strips at the instant of their prestressing.

In the case of deflections, a non-negligible effect of the CFRP strips was observed. Generally speaking, the allowable deflection was exceeded at the force 18–76% greater than for the unstrengthened specimen. The most advantageous behavior showed the unprecracked specimens (series B-II). One should note that the force associated with the allowable deflection is just the same as for the strongly strengthened precracked beam (B-I-2) and the unprecracked less strongly strengthened beam (B-II-1), which highlights the importance of the thorough bonding of cracks before strengthening.

Passive strengthening before cracking or after the bonding of cracks (beams series B-II) only to a small degree increases the cracking moment. The increase of the latter relative to the unstrengthened specimens amounted to about 20%. Whereas, the cracking moment increased spectacularly in the case of the prestressed beams (series B-III), which were characterized by about 2.5–3 times greater cracking moment than the unstrengthened beams.

### 4.2. Results of Dynamic Measurements

**Figure 20:**
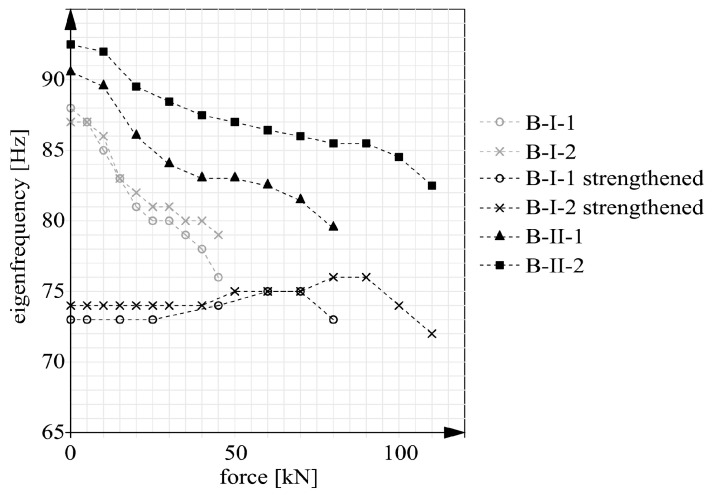
Eigenfrequencies of beams B-I and B-II versus load.

**Figure 21:**
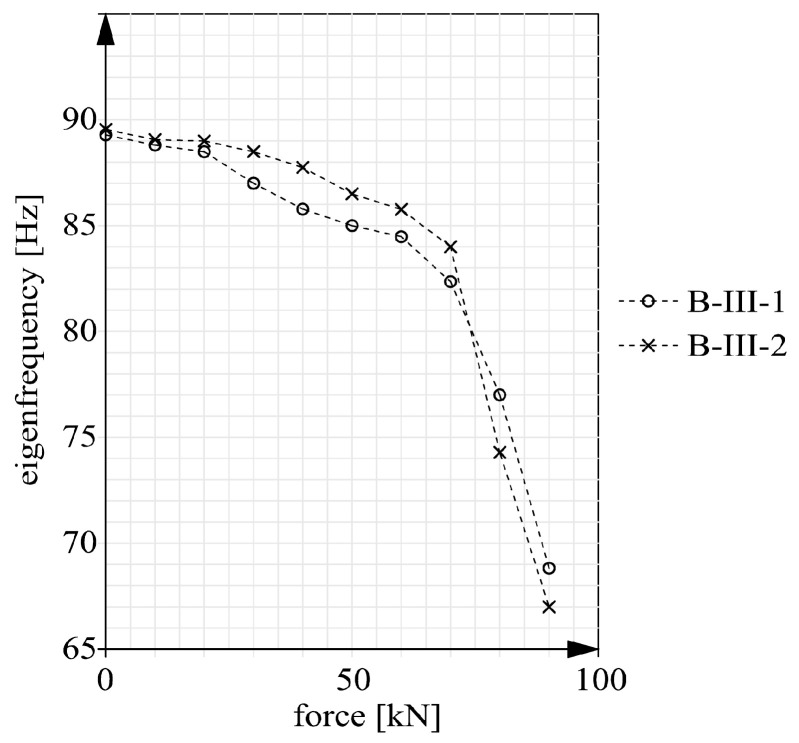
Eigenfrequencies of beams series B-III versus load.

Preliminary measurements were carried out for the debuting beams without the CFRP reinforcement. The results in the form of eigenfrequencies are presented, together with the results for the debuting strengthened beams (B-II and B-III), in Table 4. No post-strengthening results are given for beams series B-I since they were strengthened as cracked, which has an additional influence on the eigenfrequencies. The table also shows the weights of the particular beams, which will be needed in further analyses. The other eigenfrequencies measured for the beams after loading at an increasingly higher rate and unloading are presented in Figure 20 and Figure 21.

Table 4 indicates that the use of CFRP strips has no significant effect on the eigenfrequency of the uncracked member. The beam with the highest strengthening intensity (B-II-2) was characterized by a frequency gain of merely 3.9% relative to the frequency before strengthening. In building engineering this difference definitely cannot be regarded as significant. However, significant differences were observed in the frequencies of the specimens in the whole load domain and for the different types of strengthening. In the case of the unstrengthened beams (series B-I before strengthening), the eigenfrequencies are approximately constant before cracking—Figure 20. After cracking they decreased considerably. The character of the changes in eigenfrequencies depending on the load for beams with different internal steel reinforcement ratios is described in more detail in [9]. After unloading and strengthening, but before loading, the eigenfrequencies further decreased despite the fact that stiffness increasing CFRP strips had been glued to the beams. The decrease is probably due to a change in the ambient conditions [43] and a slight increase in the structure’s weight resulting from the strengthening. The loading of the cracked strengthened beams does not cause any change in the eigenfrequencies. Only when the maximum load (amounting to about 80% of the load capacity) is reached for the unstrengthened case, eigenfrequencies increase in the two beams (B-I-1 and B-I-2). The increase is proportional to the strengthening intensity. Then larger deformations arise in the tension zone, which activates the CFRP strips and their effect on stiffness. A decrease in eigenfrequencies was observed in the loading phases (from 70 kN for beam B-I-1 and from 90 kN for beam B-I-2) preceding failure. The decrease was mainly due to the appearance of inclined cracks of considerable width, near the supports where the CFRP strips were anchored.

The strengthened unprecracked beams (series B-II) behaved similarly as the unstrengthened RC beams. One can notice that the graphs for beams series B-II were similar to the ones for beams series B-I before strengthening (Figure 20). However, they were characterized by higher values and their gentler fall after cracking. This is owing to the increased cross sectional area of the external CFRP reinforcement.

The prestressed beams of series B-III were characterized by a different character of the distribution of eigenfrequencies in the load domain than the other beams (Figure 21). It should be noticed that the frequency graphs are not convex downwards as in the case of beams series B-II. There are three distinct phases in the behavior, which can be described with linear changes in eigenfrequencies. The first phase occurs before cracking and the frequencies are constant. The second phase is characterized by a linear fall and extends to the load intensity level of about 75%. The slope factor of the straight line describing the second phase amounted to 0.48. Above the load intensity level of 75% one could distinguish phase 3 with a considerable fall in eigenfrequencies. In this phase the tangent of the inclination angle of the straight line increased from 0.48 to 3.27. Because of the low prestressing force values no considerable differences between the eigenfrequencies of beams B-III-1 and B-III-2 were observed.

### 4.3. Discussion of Results

In order to compare the results of the static and dynamic measurements the flexural stiffness notions were used. Static stiffness *EI*_S_ was calculated from formula (9) on the basis of the deflections. Dynamic stiffness *EI*_D_ was calculated from formula (10) on the basis of the eigenfrequencies.

(9) $$EI_{S}=\alpha_{S}\frac{F\cdot l_{\mathrm{eff}}}{a},$$

(10) $$EI_{D}=\frac{f^{2}\cdot m\cdot l_{\mathrm{tot}}^{4}}{\alpha_{D}^{2}},$$

where: $\alpha_{S}$—a coefficient depending on the static diagram (in the considered case as for the simply supported beam: 1/48), F—the force in the given loading step, $l_{\mathrm{eff}}$—the span between the centers of the supports (3000 mm in the analyzed case), a—the midspan deflection measured in the given loading step, f—the measured first eigenfrequency, m—the beam weight per linear meter, calculated on the basis of the total weights as specified in Table 4, $l_{\mathrm{tot}}$—the beam’s overall length (3300 mm in the analyzed case) and $\alpha_{D}$—a coefficient dependent on the dynamic diagram (in the analyzed case as for the 1st eigenfrequency of the free-free beam: 3.49979).

**Figure 22:**
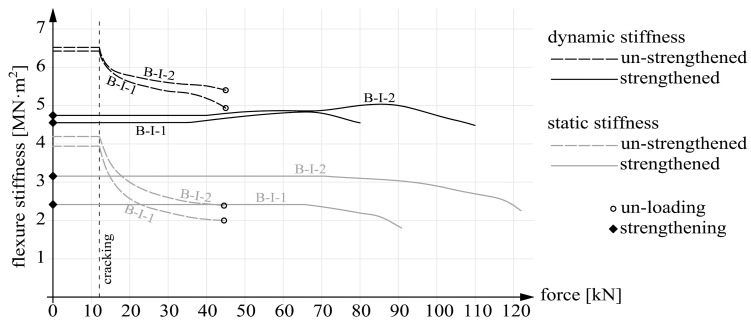
Static and dynamic stiffnesses for beams series B-I.

**Figure 23:**
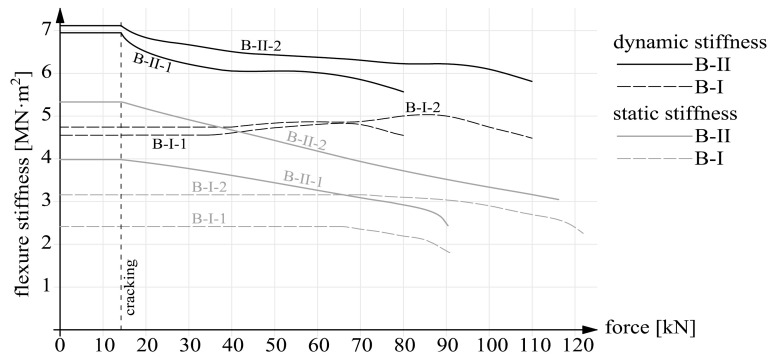
Static and dynamic stiffnesses for beams series B-II and B-I after strengthening.

**Figure 24:**
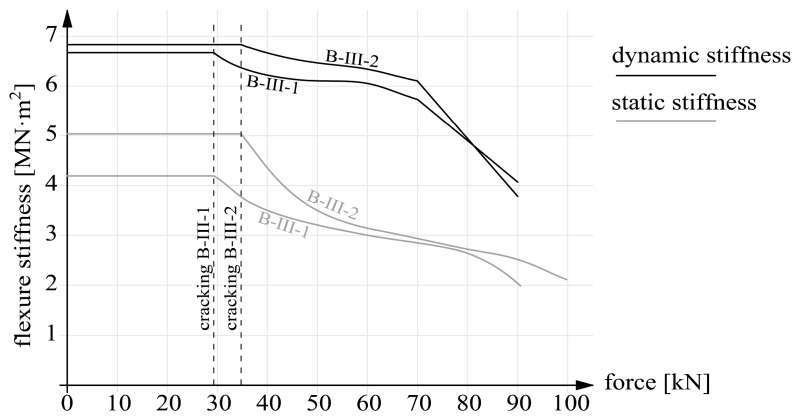
Static and dynamic stiffnesses of beams series B-III.

The calculation results, in the form of static and dynamic flexural stiffness versus load, are presented in Figure 22, Figure 23 and Figure 24. Figure 22 shows the graphs for beams B-I before and after strengthening. Figure 23 contains the graphs for beams B-II and for comparison for beams series B-I after strengthening. The graphs for prestressed beams series B-III are shown in Figure 24. The graphs show that before cracking the stiffness values were constant (Figure 20 and Figure 21). This is a simplification since a slight decrease in stiffness occurred already before the first perpendicular cracks appeared (Figure 20 and Figure 21). Considering this paper’s main theme, this simplification can be regarded as acceptable.

It should also be noted that the static stiffnesses are functions of the two variables: force F and deflection a. Whereas the dynamic stiffnesses depended solely on eigenfrequencies f. As a result, the dynamic stiffnesses were only a linear transformation of the eigenfrequencies. Thus the shapes of the dynamic stiffness graphs in Figure 22, Figure 23 and Figure 24 resemble or outright reflect the shapes of the eigenfrequency graphs shown in Figure 20 and Figure 21.

Regardless of the way in which they were strengthened, all the beams are characterized by a higher dynamic stiffness than the static one. The same trend is observed in the case of the unstrengthened RC members [9,15,16]. This is due to the fact that deflections are measured under a measurable load, while eigenfrequencies are measured at a minimal dead load intensity level and weak external impacts (random impacts, gentle excitations with the rubber hammer and air movements caused by wind and noise).

In the case of beams series B-I strengthened after cracking, the distributions of static and dynamic stiffnesses are markedly different. After strengthening the static stiffnesses increased by about 20 and 30% for the two degrees of strengthening intensity (0.224 and 0.448%). Up to the load intensity level of 65% they remained constant, then fell linearly and immediately before failure reached 70% of the initial value (after strengthening). In the case of the dynamic stiffnesses, they were found to decrease minimally after strengthening. The loading of the specimens up to about 40% of the load capacity did not result in any changes in the dynamic stiffnesses. Only when this load level was exceeded, the dynamic stiffnesses began to increase, which was connected with the activation of the CFRP strips counteracting the strains caused by bending. Above the load intensity level of 70% the dynamic stiffnesses reached their maximum and then fell mainly due to the appearance of diagonal cracks in the support zones, being also the zones of anchorage for the CFRP strips. It can be stated that in the final loading steps the dynamic stiffnesses were approximately equal to their initial values (after strengthening).

The strengthening of beams series B-II caused a slight increase in dynamic stiffnesses. According to Table 4, the increase amounted to merely 1.6 and 3.9% for respectively beams B-II-1 and B-II-2 (in comparison with the uncracked beams without the CFRP reinforcement). Additionally, in the case of beam B-II-1, the increase in static stiffness caused by strengthening can be regarded as negligible. Whereas it is noticeable in the case of beam B-II-2, amounting to over 30%. One should note that beams series B-II were not loaded before strengthening. That is why unstrengthened beams series B-I were adopted as the reference. After cracking the static and dynamic stiffnesses differ markedly in their course in the load domain. The dynamic stiffnesses change slightly and the course of the changes is nonlinear. In the final phase of loading the dynamic stiffnesses amount to about 80 and 85% of the initial values for beams B-II-1 and B-II-2, respectively. After cracking the static stiffnesses are characterized by a quasi-linear fall and at the instant preceding failure they amount to about 63% and 57% of their initial value for beams B-II-1 and B-II-2, respectively. A comparison of the stiffness of beams series B-I and B-II shows that despite the same degrees of strengthening in the case of pairs B-I-1 and B-II-1; B-I-2 and B-II-2, the beams behaved markedly differently. The graphs prove how important, besides the use of external strengthening, the injection of cracks can be. It can considerably improve the performance of beams.

A separate figure (Figure 24) shows stiffness graphs for prestressed beams series B-III. In the case of dynamic stiffnesses before cracking, the effect of the strengthening was found to be negligible (an increase by 1.6 and 1.8% for respectively beam B-III-1 and B-III-2). In the case of static stiffnesses, a significant increase, amounting to about 25%, was registered for only beam B-III-2. Similarly as in the case of series B-II, the increment in static stiffnesses was related to the static stiffnesses of unstrengthened beams series B-I. As their cracking resistance was increased by prestressing, beams series B-III were characterized by constant stiffnesses in about 1/3 of the loading range. After cracking the dynamic stiffnesses of the two beams fell as a result of loading. The fall is gentler up to the load level of 70 kN for both the beams. At this level the graph inflects and the stiffnesses fall linearly until failure. Before failure they reached about 60% of the initial values, similarly for both the beams. The static stiffnesses after cracking were characterized by a nonlinear fall. At the load intensity level of about 90% the graphs had inflexion points. This was connected with the softening of the concrete in the midsection of the beam. One should note that in the load interval of 55-80 kN static stiffnesses ere very similar for the two beams of series B-III.

## 5. Conclusions

The following conclusions emerged from the above research:

- The dynamic stiffnesses were higher than the static ones for all the tested beams, due to, i.a., the minimal load intensity level during the investigation of eigenfrequencies, which depend on the vibrating mass as regards not only inertia, but also stiffness;
- The strengthening of the beams results in a much larger increment in static stiffnesses than in dynamic stiffnesses (this is particularly visible in the beams with higher strengthening intensity, i.e., B-I-2, B-II-2 and B-III-2); the numerical values of the increments are given in Table 5;
- Within a single series the dynamic stiffnesses of the beams differing in their strengthening parameter (the cross sectional area or the prestressing force) did not differ as much as the static stiffnesses, which means that increased strengthening intensity did not translate so much into dynamic stiffness as into static stiffness;
- In the case of (precracked) beams series B-I, the static and dynamic stiffnesses after strengthening considerably differed qualitatively and quantitatively from each other; the negative effect of the cracks not repaired before strengthening on the structure’s static and dynamic responses is clearly visible.

The tests have shown that the structure’s responses, i.e., experimental displacements and eigenfrequencies, could lead to different results in the form of stiffness. One should bear this in mind when carrying out laboratory and in-situ tests. Therefore when diagnosing structures it is recommended not to limit oneself to the registration of only one type of the structure’s response.

It should be emphasized that the external strengthening of beams with CFRP strips modifies the static and dynamic stiffnesses, particularly their distributions in the load domain. Despite the relatively small cross-sectional area of CFRP strips and their *E*-modulus close to the *E*-modulus of steel, their effect on the static and dynamic stiffnesses cannot be neglected.

## Figures and Tables

**Table 1:** Strength and deformation properties of materials used in tests.

Properties of Concrete			Properties of Steel			Properties of CFRP Strips		
*f*_cm_	*E*_cm_	*f*_ctm,spl_	*f*_ym_	*E*_sm_	*f*_yt_	*f*_f_	*E*_f_	*ε*_fu_
(MPa)	(GPa)	(MPa)	(MPa)	(GPa)	(MPa)	(MPa)	(GPa)	(%)
50.8	27.3	6.7	558	195	617	3500	210	1.7

**Table 2:** Comparison of tested beams.

No.	Type of Strengthening	Beam Denotation	CFRP Strip Cross Section *A*_f_ (mm^2^)	Strengthening Parameter
1	No strengthening	B-0	-	-
2	Passive strengthening after preloading beam to 80% of its strength	B-I-1	1.4 × 60 = 84	0.224%
3		B-I-2	1.4 × 120 = 168	0.448%
4	Strengthening of debuting beam	B-II-1	1.4 × 60 = 84	0.224%
5		B-II-2	1.4 × 120 = 168	0.448%
6	Active strengthening (prestress) of debuting beam	B-III-1	1.4 × 50 = 70	40 kN
7		B-III-2	1.4 × 50 = 70	75 kN

**Table 3:** Forces, bending moments and deflections for cracking and failure.

No.	Beam	Cracking			Failure		
		Force *F*_cr_	Bending Moment *M*_cr_	Deflection *a*_cr_	Force *F*_R_	Bending Moment *M*_R_	Deflection *a*_R_
		(kN)	(kNm)	(mm)	(kN)	(kNm)	(mm)
1	B-0	11.15	8.36	1.943	57.41	43.06	32.031
2	B-I-1	12.22 ^1^	9.17 ^1^	1.756 ^1^	90.99	68.24	28.606
3	B-I-2	12.24 ^1^	9.18 ^1^	1.647 ^1^	121.92	91.44	30.675
4	B-II-1	14.34	10.76	2.036	90.23	67.67	20.754
5	B-II-2	14.46	10.85	1.527	116.01	87.01	21.443
6	B-III-1	29.38	22.04	3.867	90.65	67.99	25.873
7	B-III-2	34.93	26.20	3.725	99.90	74.93	27.066

^1^ Values for RC beams (before strengthening). Nomenclature in Table 3: *F*_cr_—cracking force, *M*_cr_—cracking moment, *a*_cr_—deflection associated with cracking, *F*_R_—maximum force (strength), *M*_R_—maximum moment (strength), *a*_R_—deflection associated with ultimate strength.

**Table 4:** Weights and eigenfrequencies of debuting beams.

No.	Beam	Weight	Eigenfrequency		
			Before Strengthening	After Strengthening	Relative Increment
		(kg)	(Hz)	(Hz)	(%)
1	B-I-1	291	87.0	-	-
2	B-I-2	295	88.0	-	-
3	B-II-1	300	89.1	90.5	1.6
4	B-II-2	290	89.0	92.5	3.9
5	B-III-1	288	88.6	89.3	1.0
6	B-III-2	294	88.7	89.5	0.9

**Table 5:** Increments in stiffness for particular beams.

No.	Beam	Increment in Static Stiffnesses	Increment in Dynamic Stiffnesses
		(%)	(%)
1	B-I-1	22 ^1^	0 ^1^
2	B-I-2	32 ^1^	0 ^1^
3	B-II-1	0 ^2^	3.2 ^2^
4	B-II-2	32 ^2^	8.0 ^2^
5	B-III-1	3.1 ^2^	1.6 ^2^
6	B-III-2	25 ^2^	1.8 ^2^

^1^ increment between the final step of loading the unstrengthened beam and the first step of loading the strengthened beam; ^2^ increment for the debuting beam, resulting from strengthening (the static stiffnesses were related to unstrengthened beams series B-I).

## Data Availability

Data is contained within the article.
